# Enhancing the Solubility of Indomethacin: A Breakthrough with Cocrystal Formation

**DOI:** 10.3390/ph18111610

**Published:** 2025-10-24

**Authors:** Hugo Pardo, Víctor Guarnizo-Herrero, Borja Martínez-Alonso, Mª Ángeles Peña Fernández

**Affiliations:** Department of Biomedical Sciences, Faculty of Pharmacy, University of Alcalá (UAH), Campus Universitario, Crta. Madrid—Barcelona km. 33.600, Alcalá de Henares, 28771 Madrid, Spain; hugo.pardo@edu.uah.es (H.P.); victor.guarnizo@uah.es (V.G.-H.); borja.martineza@uah.es (B.M.-A.)

**Keywords:** indomethacin, solubility, cocrystals

## Abstract

**Background/objectives:** Pharmaceutical cocrystals have emerged as a promising strategy to enhance the solubility and bioavailability of poorly water-soluble drugs. Indomethacin (IND), classified as a Biopharmaceutics Classification System (BCS) Class II drug, exhibits low solubility but high permeability. This study aims to develop a synthesis method, evaluate cocrystal solubility/stability and the physicochemical properties of the pure components, and describe cocrystal solubility using a mathematical model. **Methods:** Cocrystals were synthesized via solvent evaporation, using ethanol, methanol, and ethyl acetate. The pure components, IND and benzoic acid (AcBz) were dissolved in each solvent and maintained in a thermostabilizer for 24 h. Cocrystal formation was confirmed by UV-V spectroscopy, differential scanning calorimetry (DSC), and infrared (IR) spectroscopy. **Results:** The results showed that the solubility of the cocrystals decreased with increasing benzoic acid concentration. Mathematical modelling revealed that solubility can be expressed as the product of the solubilities of the individual components and the stability constant of the solution complex. Among the solvents tested, ethanol exhibited the highest solubility and equilibrium constant (K_eq_) for IND–AcBz cocrystals, suggesting a greater molecular affinity and enhanced cocrystal formation. **Conclusions:** These findings demonstrate that the formation of the novel INDAcBz cocrystal significantly enhances Indomethacin solubility and thermodynamic stability. These results validate benzoic acid as an effective coformer and establish phase solubility diagrams (PSD) as predictive tools for rational cocrystal design, supporting the future development of optimized pharmaceutical formulations.

## 1. Introduction

Cocrystals, a distinct class of crystalline materials with two or more molecular entities, are important in green chemistry due to their ability to improve drug delivery systems. Most new active pharmaceutical ingredients (APIs) have poor aqueous solubility, and approximately 60–70% are classified as BCS classes II and IV. Low water solubility leads to poor bioavailability, restricting pharmaceutical formulation viability. Variable pH conditions along the gastrointestinal tract further complicate drug solubility and absorption, challenging consistent efficacy and safety. Cocrystal design is a promising strategy for optimizing the properties of pharmaceutical materials. Cocrystals allow the modulation of mechanical properties, hygroscopicity, stability, and solubility, depending on the coformer selected for formation. This enhances the rational design of materials with adjustable features. Various methods have sought to improve poorly water-soluble APIs, each with advantages and limitations. Selecting an appropriate strategy involves considering the API’s properties, excipients, process, and dosage form [1,2,3,4,5,6,7,8,9].

Cocrystals are composed of two or more solid components held together by noncovalent interactions (e.g., hydrogen bonds). Cocrystals offer a stable crystalline form that enhances the physicochemical properties of APIs without requiring additional excipients; they improve solubility, stability, and bioavailability, while preserving pharmacological activity [7].

### 1.1. Cocrystal Intermolecular Interactions

Non-covalent forces like hydrogen bonding, π-π interactions, and van der Waals forces contribute to cocrystal formation. The Cambridge Structural Database (CSD) is a key tool for understanding crystal engineering through non-covalent supramolecular bonds. Bonds are formed primarily by carbonyl oxygens and aromatic nitrogens, while donors follow: COOH > NH >> R-OH. Competition between homosynthon and heterosynthon molecules determines the resulting bonds [3,4,5].

### 1.2. Crystallization as a Tool for Optimizing Drug Properties

Strategies to optimize the physicochemical properties of cocrystals started 25 years ago. In the last decade, the synthesis of pharmaceutical cocrystals has gained great interest, with a wide variety of drugs currently on the market. Cocrystals modify API properties by altering the solid-state structure. Forms include salts, polymorphs, hydrates, solvates, and cocrystals. Characteristic crystallinity, melting point, solubility, dissolution rate, and stability enable the prioritization of promising pharmaceutical forms [1,2,3].

Melting point and stability—determined via DSC—are key indicators of purity and properties and are inversely correlated with solubility and vapour pressure [2,4,7,10].

### 1.3. Solubility and Methods of Cocrystals Formation

Solubility is a key parameter in drug development and can be expressed as thermodynamic solubility, dissolution rate, and intrinsic solubility. Cocrystal engineering is often explored to improve poorly soluble APIs [4].

Formation of pharmaceutical cocrystals uses techniques including solid (grinding, sonication) and solvent-based methods (solution crystallization, evaporation). Selection of optimal solvent is crucial, as it affects interactions and cocrystallization [2]. Solution synthesis is based on the combination of equimolar amounts of API and coformer in a suitable solvent [1]. Successful crystallization depends on supersaturation, differences in solubilities and solvent temperature conditions [1,4,9,11].

Solvent evaporation is one of the most widely used techniques for obtaining cocrystals, due to its high efficiency and the possibility of generating specific crystalline forms [8,9,10,11,12,13,14,15,16,17,18,19,20]. The procedure consists of dissolving the API and the coformer in a common solvent, in appropriate stoichiometric proportions. Subsequently, the solution is left at room temperature, usually in a flow hood, until complete evaporation of the solvent. During this process, nucleation and crystal growth occurs, favouring the formation of the desired cocrystal [12,13,14,15,16,17,18,19,20,21]. A representative example of this methodology is found in the study of Ji et al.’s study, where cocrystals of caffeine and malonic acid were obtained by slow solvent evaporation [4,22,23,24,25,26,27,28,29,30,31].

### 1.4. Theorical Solubility

Understanding the solubility behaviour of cocrystals in component solutions is a fundamental challenge. This study models complex formation and the influence of intermolecular interactions (hydrogen bonding, van der Waals, electrostatics) on solubility. In 1:1 stoichiometric cocrystals, each component forms a saturated solution, expressed as the cocrystal’s molar solubility [32].

Predicting and explaining cocrystal solubility behaviours heavily relies on phase diagrams and triangular composition diagrams. The solubility phase diagram and triangular solubility diagrams are essential tools for mapping and comparing cocrystal stability. Phase solubility diagram (PSD) analysis clarifies crystallization mechanisms and is valuable for scale-up optimization. PSDs display API concentration as a function of coformer concentration, defining stability zones of cocrystals and leading to crystallization [9,33].

#### 1.4.1. Determination of the Phase Solubility Diagram (PSD)

Representation of the data in a PSD defines the stability regions of the cocrystal and the individual components, and the result has led to the development of the evaporative crystallization method [34].

**Figure 1:**
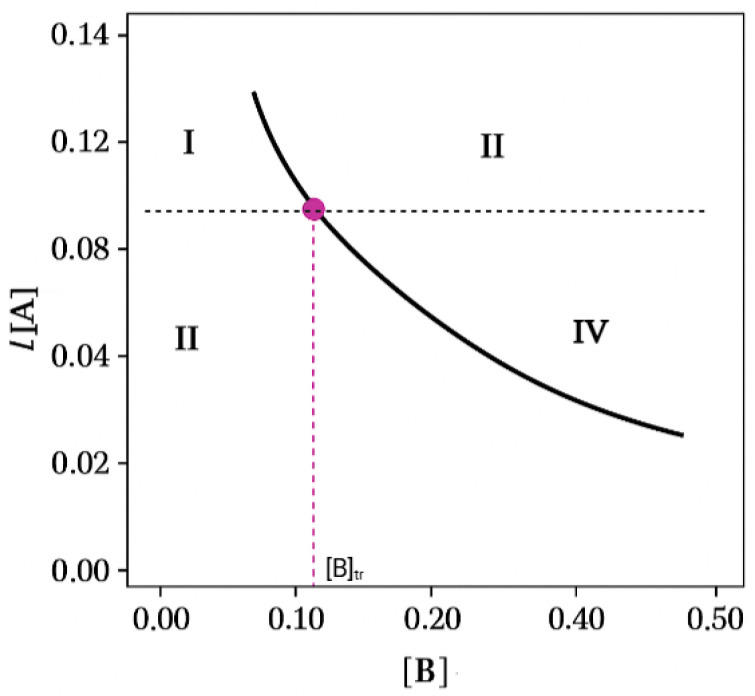
Theorical graph of PSD simulation, representing effect of ligand concentration on the solubility of cocrystal AB. The black dashed line, compound A, represents the solubility of a single component. And the purple dashed line corresponds with the transition ligand concentration [B]_tr_ point, where the solubility of A is equal to solubility of AB.

The theoretical graph in Figure 1 shows four regions of thermodynamic stability, labelled with Roman numerals, for the different dissolution or crystallization phases of the multicomponent phase system. For this experiment, in region I, the IND-AcBz cocrystal is unsaturated, with respect to AcBz, and is less soluble than the active ingredient, and only the active ingredient is supersaturated, being more soluble. Only IND crystals are formed in the solid phase. On the other hand, in region II, both the active principle and the coformer are supersaturated. This is the region in which IND and IND-AcBz crystals can form together. In contrast, both are unsaturated in region III. No crystals form in this region. Likewise, region IV is characterized by having mostly supersaturated cocrystals with respect to API, which is unsaturated. For all these reasons, region IV is the thermodynamically stable zone, and where there is a greater potential for the synthesis of cocrystals. Identifying the conditions of these zones is what must be sought in the scaling-up process in the synthesis of cocrystals [34].

The PSD reveals that the solubility of the cocrystal decreases progressively as the concentration of the coformer increases (benzoic acid). This effect is consistently observed in all solvent systems. Benzoic acid acts as a key modulator of the solubility equilibrium, influencing phase stability. In an incongruent system, where the API exhibits lower solubility than the coformer, this promotes the formation of the thermodynamically stable cocrystal, whereas congruent systems, where both components display comparable solubilities, facilitate the generation of 1:1 cocrystals, due to the balanced molecular interactions in the solutions [9,32,35,36].

#### 1.4.2. Determination of the Triangular Diagram Phases (TPD)

In the cocrystallization experiment, the composition of the solution phase (S: solvent, A: API, B: coformers) critically influences the formation 1:1 stoichiometric cocrystal (AB) [3]. Sometimes, multicomponent systems fail to yield cocrystals due to differences in solubility, intermolecular interactions, or thermodynamic stability, resulting in precipitation of a single component, polymorph, or undesired crystalline form. Ternary (solute–solute–solvent) diagrams are essential tools for interpreting equilibrium phases, guiding cocrystal discovery and API nucleation, and defining crystallization pathways and thermodynamic stability [9,36].

**Figure 2:**
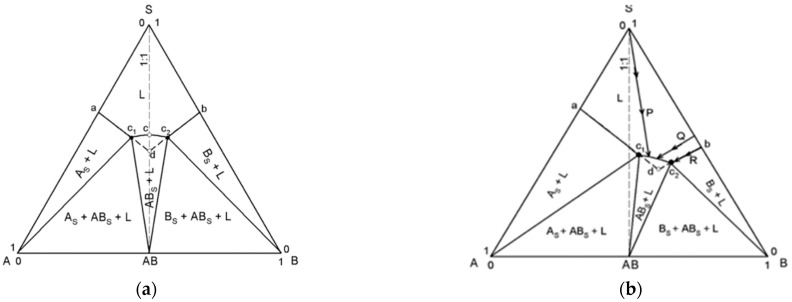
(**a**). Schematic theorical TPD for 1:1 cocrystal, representing the similar solubility of the compounds. (**b**). Schematic theorical TPD for 1:1 cocrystal, representing different solubility of the compounds.

The key to the composition of a high-purity crystal lies in understanding the equilibrium of the ternary diagram. TPDs are influenced by the relative solubility of the two components. If the solubilities are similar, it is seen in Figure 2a, while Figure 2b shows two components with different solubilities. Slow evaporation of the 1:1 cocrystal can lead to the formation of the cocrystal from a mixture of the cocrystal with one of the individual components. This all depends on where the crystallization phase is located and what the concentration of the solution is; whether it is in the mixed phase or in the single phase [8].

The position of the solid region in a cocrystal system depends largely on the nature of the solvents. Therefore, it is expected that the choice of solvent, where the API and the coformer have similar solubility, will generate symmetrical phase diagrams. The asymmetry of the diagrams becomes more marked as the differences in solubilities of the API and coformer in the solvent increase. These are reflected in other cocrystal systems, such as carbamazepine-nicotinamide [36].

These advances represent a significant step toward the rational design of pharmaceutical cocrystal aimed at enhancing the efficacy and bioavailability of APIs. This study reports, for the first time, the successful crystallization and characterization of a new indomethacin–benzoic acid (IND–AcBz) cocrystal. In this context, the use of benzoic acid, a pharmaceutical acceptable compound, as a coformer introduces a novel approach, due to its dual role as a stabilizing agent and solubility modulator. References [32,33,34,35,36] provide new insights into the design of the pharmaceuticals-relevant cocrystal. These findings pave the way for future investigation into the in vivo performance and modelling of the multicomponent cocrystal system.

## 2. Results and Discussion

**Figure 3:**
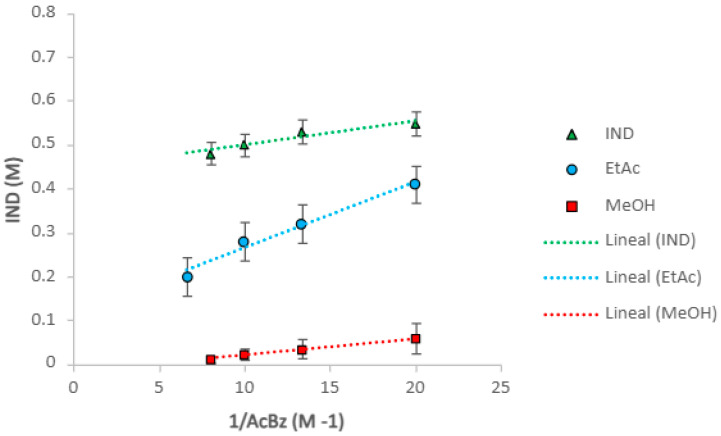
Plot of [IND]_T_ in equilibrium with (IND + AcBz) cocrystal, against 1/AcBz at T = 273.15 K.

The representation of [IND] as a function of 1/[AcBz] produces a linear representation. The result is shown in Figure 3, where a good linearity is observed. The equations of the line and the model parameters, K_sp_ and K_11_, are shown in Table 1. This equation predicts where the complex is formed in the solution, and which concentrations are optimal for IND-AcBz solubility. The values of K_sp_ correspond to the slope, while K_11_ is determined by the intercept with the axis, as reflected in Table 2. According to the definition, K_sp_ depends on the solvent used and is proportional to the solubility of the cocrystal in the solvent. Furthermore, K_sp_ is subject to temperature, and increases as a function of individual solubility values, leading to higher K_sp_ values.

The solubility of the IND-AcBz cocrystal and of the individual components in the different solvents is reflected in Table 2.

The solubility of the cocrystal varies as a function of the solvent, as follows: ethyl acetate > ethanol > methanol. IND is the least soluble component of the two cocrystal formers in these solvents, and the solubility of the more thermodynamically stable polymorph γ ranks in the following order: ethyl acetate > methanol > ethanol, following a similar order to the cocrystal. The solubility ratio of the individual cocrystal components is important for considering the conditions of crystallization and isolation of the crystal in the evaporation method is reflected in Table 3. This behaviour can be anticipated with the solubility product, following the reaction equilibrium of Equation (1), assuming that the solution complex is 1:1 [34].

(1) $${IND-AcBz}_{solid}\;\overset{Ksp}{\Leftrightarrow }\;{IND}_{solution}+{AcBz}_{solution}$$

where the solubility product is as follows:

(2) $$K_{sp}=\left[IND\right]\;[AcBz]$$

The solubility products are calculated through the slopes and points of the total IND concentration in the solution, versus the inverse of the total [AcBz] concentration in the solution, according to Equation (3):

(3) $$[IND]=\frac{K_{sp}}{\left[AcBz\right]}$$

While the equilibrium of the complex solution is written as Equation (4):

(4) $${IND}_{solution}+{AcBz}_{solution}\;\overset{K11}{\Leftrightarrow }{IND-AcBz}_{solid}$$

where K_11_, the complex constant, is given by Equation (5):

(5) $$K_{11}=\frac{\left[IND-AcBz\right]}{\left[IND\right][AcBz]}=\frac{\left[IND-AcBz\right]}{Ksp}$$

K_11_, on the other hand, is defined as the equilibrium constant for complex formation in the solution, which is determined from the solubility of the cocrystal and is a function of the concentration of the coformer. At the same time, the lower the solubility of the co-crystal and the components, the greater the predisposition to form complexes in the solution is [32,37]. The presence of non-zero values suggest the possible non-existence of a 1:1 congruent system in the system studied. This may be because the solute–solute interaction is preferred over the solute–solvent interaction. In this study, a large difference is observed between the different solvents, with high values for ethanol and much lower, but practically similar, values for ethyl acetate and methanol. Although in methanol, the Y intercept value is not statistically significantly different from zero, the K_11_ values were not calculated, because it presented lower values compared to the associated standard error. High K_11_ values suggest a higher stability of the API and coformer complex. This means that more of the API will remain in the solution as part of the complex [32,34,38].

Where the solubility of the cocrystals is expressed as the following:

(6) $${\left[IND\right]}_{T}=\frac{Ksp}{{\left[AcBz\right]}_{T}-K_{11}Ksp}+K_{11}Ksp$$

If the value of K11Ksp is << [AcBz], then we obtain the following:

(7) $${\left[IND\right]}_{T}=\frac{Ksp}{{\left[AcBz\right]}_{T}}+K_{11}Ksp$$

This equation predicts when it is a solution complex, and which is the optimum zone of solubility of the IND-AcBz cocrystal, by the constant values (the product of K_sp_ and K_11_). Table 2 shows the calculated values of the K_sp_ slopes and the *Y*-axis intercept values, according to Equation (7). The solubility curves of the cocrystals obtained with this equation are shown in Figure 3. The K_sp_ values follow the same relative order as the solubility of the cocrystals, ethyl acetate > ethanol > methanol.

**Figure 4:**
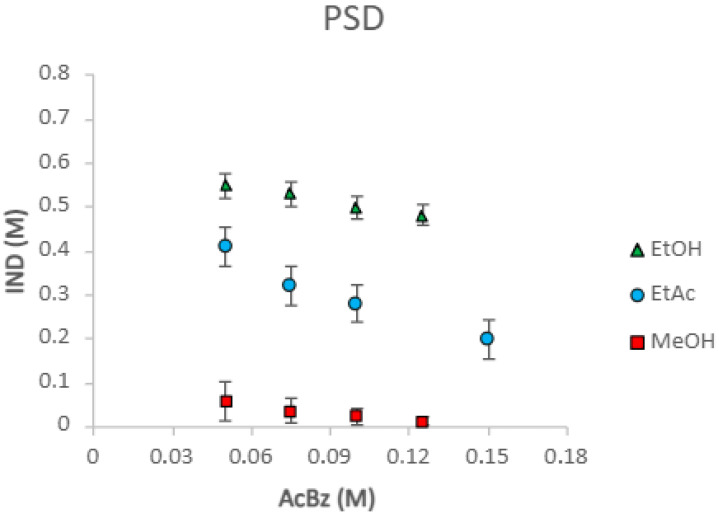
Solubility of (IND-AcBz) cocrystal in ethyl acetate, methanol, and ethanol, as a function of benzoic acid concentration. The solid line represents the solubility prediction according to Equation (3), using the K_11_ and K_sp_ values from Table 1.

Figure 4 shows a greater predisposition to form solution complexes in solvents where the solubility of the components and of the cocrystal are lower, due to the greater tendency for solute–solute interactions to occur, relative to solute–solvent. In addition, the K_11_ determined from the above equations can be used to predict the solubility of the IND [21].

(8) $${\left[IND\right]}_{T}={\left[IND\right]}_{o}+\frac{K_{11}{\left[IND\right]}_{o}{\left[AcBz\right]}_{T}}{1+K_{11}{\left[IND\right]}_{o}}$$

where [IND]o is the solubility of IND in the pure solvent, without coformer. The solubility of IND in pure solvents and IND in the solution with benzoic acid is slightly different.

Preliminary studies of the solubility measurements of the individual crystal components with IND in saturated AcBz solutions, to achieve the solubility of the cocrystal, S, show the transformation of IND into a cocrystal in Figure 3. The solubility measurement of pure IND in the solvent and the predicted solubility as a function of the cocrystal concentration of AcBz solutions are the result of the solution complexing. The K_11_ values from Table 2 are used in Equation (7) to predict the solubility of IND in saturated AcBz solutions. In the experiment of Vasoya et al. [39], the solubility of ketoconazole and its cocrystals with fumaric and succinic acid was studied, where solubility was observed to be affected as a function of pH ranges, but also attributed to interactions in the solution with a high K_11_ value. Thus, the solubility of API in the presence of cocrystals is not only dependent on the solubility constant (K_sp_), but it also depends on the formation of complexes in the solution (K_11_) [39].

### 2.1. Eutectics Concentrations of the IND-AcBZ Cocrystal

For the crystallization process, it is important to know the eutectic point (cocrystal–API and cocrystal–coformer). The easiest way to obtain the eutectic point is to determine the zone in which the cocrystal and the API coexist in equilibrium in the solution, which is why the solubility of the API is usually lower than that of the coformer. This happens in incongruent cocrystal saturation systems. Under ideal behavioural conditions, the intrinsic solubility of the cocrystal can be predicted from the eutectic concentrations of the crystal components.

(9) $$S_{cocrystal}=\sqrt{{\left[IND\right]}_{eu}{\left[AcBz\right]}_{eu}}$$

The calculation of the solubility in the different solvents, through Equation (9), gives values very similar to the pure solubility values. With this model, the solubility for congruent systems can be determined with adequate precision. For incongruent systems, on the other hand, the solution equilibrium cannot be reached. However, the model can be used to estimate the solubility at equilibrium, since it cannot be determined experimentally in comparison to congruent systems [34]. K_eu_ has been found to be a good indicator of cocrystal solubility and stability. The K_eu_ (observed) can be calculated from the eutectic concentrations of the components by the following equation:

(10) $$K_{eu}=\frac{{\left[AcBz\right]}_{eu}}{{\left[IND\right]}_{eu}}$$

It has been observed that when K_eu_ values < 1, it is a congruent saturation system, while if K_eu_ > 1, it is an incongruent saturation system in the respective solvents. For the IND-AcBz cocrystal, K_eu_ < 1 in the solvent’s methanol and ethyl acetate and > 1 in ethanol. This means that in methanol and ethyl acetate, it is congruent, and in ethanol, it is incongruent. It is observed that the solubility of the coformer is higher than the API if the cocrystals are in an incongruent system, as shown in the article on cocrystals of indomethacin–saccharin by Min- Jeon Lee et al. [37].

**Figure 5:**
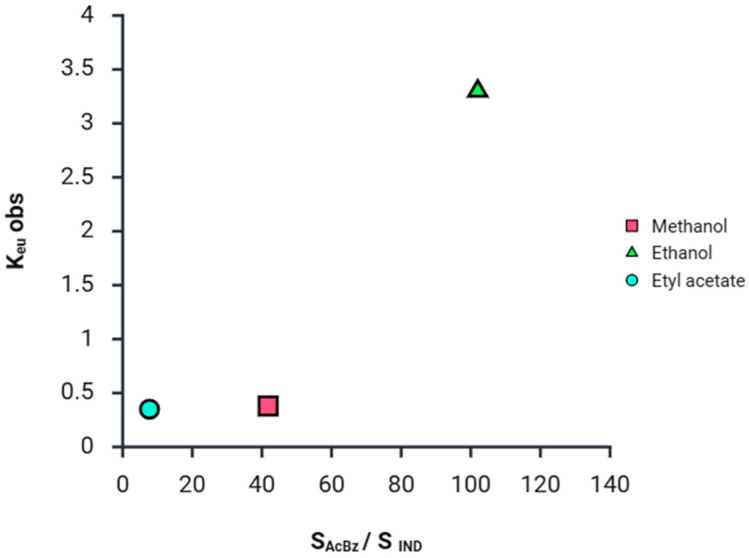
Relationships between the observed eutectic constant (K_eu_ obs) and the solubility ratio of the coformer to the API (S_AcBz_/S_IND_) in different solvents.

The K_eu_ is studied to compare the relationship between the cocrystal components and the solubilities of the cocrystals in different solvents. Figure 5 shows that the solubility of AcBz is lower than that of IND in ethyl acetate and methanol, respectively, because of the low K_eu_ values of the cocrystals in these solvents. These low K_eu_ values indicate a lower capacity to form eutectic mixtures. However, this trend is not followed in ethanol, where the IND-AcBz cocrystal solubility and K_eu_ value are higher. In summary, the concepts explained in the paper are directly related to the scaling of the cocrystal formation process. By developing the solubility models proposed above, we demonstrate the importance and practical application of the eutectic values and the K_eu_ value of cocrystals in different solvents. The eutectic points in the ternary solubility diagrams are very useful in establishing stability and solubility limits. In incongruent cocrystal saturation systems, the Keu values are the closest point measured at equilibrium.

While cocrystal solubility is a useful operational variable, its value depends on the solution composition of the cocrystal. The K_sp_ from the cocrystal solubility in pure solvents can be used to estimate the cocrystal solubility dependence of the coformer. In this study, the different solvents that are employed show that for the IND-AcBz cocrystals, a higher solubility and K_eu_ ratio is observed in the ethanol solvent, which suggests a greater molecular affinity and propensity towards the formation of cocrystals.

### 2.2. Ternary Diagrams

Cocrystal formation is obtained upon reaching a saturation concentration in the solutions. Between the two concentrations, the solution is concentrated with respect to the two solid phases: IND over cocrystal at c1 and AcBz over cocrystal at c2. The solution in the region c1–c2 is supersaturated with cocrystal, with respect to the individual components. According to the graph, only cocrystal formation is possible in this region. The line a-c1 represents that the solubility of IND in the solution increases with the increasing AcBz concentration. For the representation of the solubility behaviour, it is necessary to convert the individual concentrations (IND/S and AcBz/S) into total solution composition (IND/(IND + AcBz + S)) with the appropriate units, mole fraction, or mass percentage [9]. For the representation of the solubility behaviour, it is necessary to convert the individual concentrations (IND/S and AcBz/S) into total solution composition (IND/(IND + AcBz + S)), with the appropriate units, mole fraction, or mass percentage [9]. In this case, cocrystals can be generated in the solvent by using stoichiometric proportions of the API–coformer.

**Figure 6:**
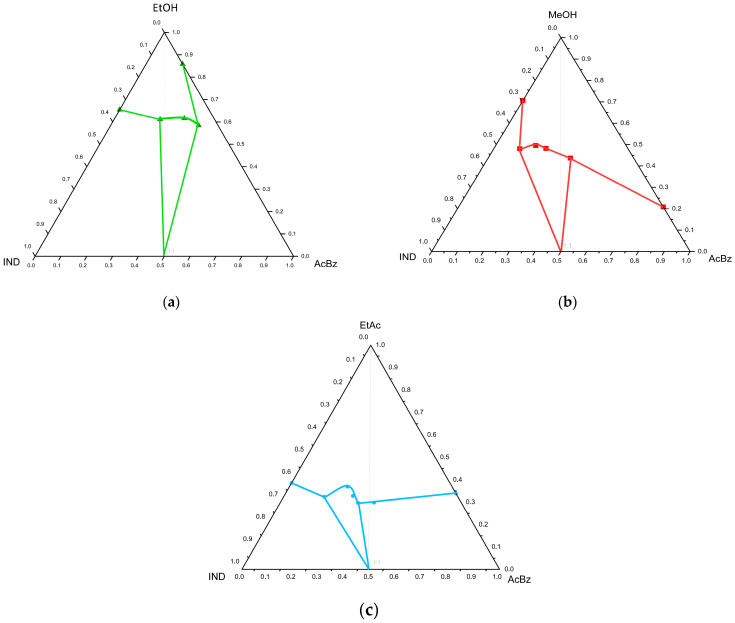
Ternary phase diagrams for the following: IND + AcBz + EtOH (**a**), IND + AcBz + MeOH (**b**), and IND + AcBz + EtAc (**c**) at 298.15 K.

Cocrystal formation is obtained upon reaching a saturation concentration in the solutions. Between the two concentrations, the solution is concentrated with respect to the two solid phases: IND over cocrystal at c1 and AcBz over cocrystal at c2. The solution in the region c1–c2 is supersaturated with cocrystal with respect to the individual components. According to the graph, only cocrystal formation is possible in this region. The line a-c1 represents that the solubility of IND in the solution increases with increasing AcBz concentration. In this representation, three distinct phase regions can be distinguished. Firstly, the cocrystal-forming region was differentiated, mainly where AcBz exceeds IND, and the formation of stable IND-AcBz cocrystals is observed. The asymmetry of the graph suggests that a higher proportion of AcBz is needed to stabilize the cocrystal. On the other hand, the second region shown is the one corresponding to the solid precipitation regions. They are characterized by an excessive, solubility-limiting amount of one of the IND or AcBz components that causes solid precipitation. And finally, there is the region formed by high amounts of solvent: no solid formation is observed. This region is relevant for controlled crystallization processes. The ternary diagrams generated from the experimental data are shown in Figure 6a–c. This region is relevant for controlled crystallization processes. The ternary diagrams generated from the experimental data are shown in Figure 6a–c. It is observed that the experimental points tend to concentrate on the 1:1 stoichiometry line in the case of the EtOH and MeOH cocrystals, unlike ethyl acetate, as shown in Figure 6a–c. Therefore, it is confirmed that those cocrystals that are on the stoichiometry line present a defined and stable proportion under the conditions studied. In the TPD of the solvent ethyl acetate, a tendency is observed to shift away from the stoichiometric line, deviating toward an excess of IND. This suggests that the stability range of the cocrystal is narrow, as confirmed by the marked differential solubility system.

The TPD study confirms that API–coformer cocrystal formation occurs in a nearly stoichiometric 1:1 ratio, with a defined stability range against individual excesses of each component. The interpretation of solid–liquid equilibrium regions demonstrates that the relative solubility of the constituents is a determining factor for the stability and extent of the cocrystal domain. These findings provide solid experimental evidence supporting the selection of optimal crystallization conditions and the scale-up of cocrystal production, thus contributing to the thermodynamic understanding of their formation.

As discussed above, if it is an incongruent system, solubility, characterized by a lower apparent solubility of IND compared to the coformer in ethyl acetate, is compared to the coformer (benzoic acid). The interaction in the solution plays an important role in modelling the solubility of the cocrystal [35,36]. The results of the triangle diagram show that the formation of IND-AcBz cocrystals is critically dependent on the composition of the mixture. The crystallization region, requiring an excess of AcBz, reflects molecular interactions, such as hydrogen bonds, between Figure 6a–c.

### 2.3. Physical Properties of Cocrystals

#### 2.3.1. Differential Scanning Calorimeter (DSC)

**Figure 7:**
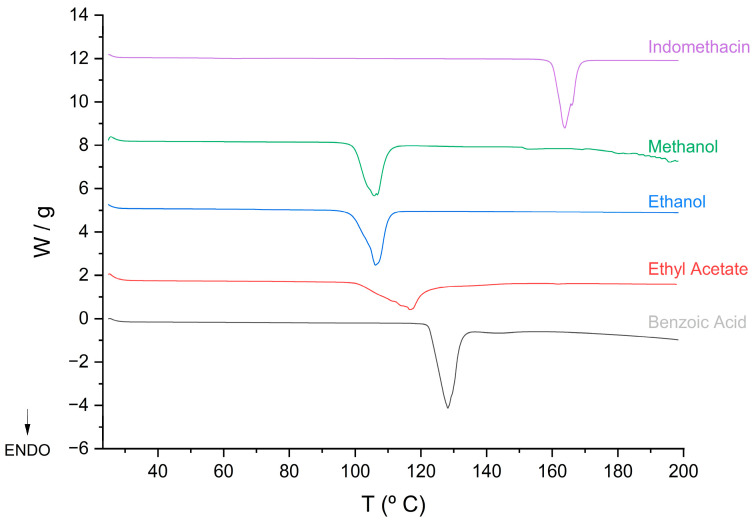
DSC curves of IND, AcBz, and the cocrystal in the diferent solvents (EtOH, MeOH, EtAc).

The characterization of IND, benzoic acid, and the newly obtained crystalline form IND-AcBz was performed using differential scanning calorimetry (DSC). This technique was employed to gain insights into the thermal behaviour of each compound, including melting points, phase transitions, and potential interactions between components. The objective was to establish a correlation between their physicochemical properties and thermal stability, which could provide valuable information for understanding the formation and stability of the new crystalline phase [40]. Figure 7 shows the results for IND, which exhibits a single endothermic event, corresponding to melting at a T_peak_ of 162.69 °C and an enthalpy of fusion (ΔH_fusion_) of 91.051 J·g^−1^. This indicates that IND is present in its γ polymorphic form. Benzoic acid shows a peak at an T_peak_ of 122.75 °C, with a ΔH_fusion_ of 124.20 J·g^−1^. The thermogram of the IND-AcBz cocrystal displays a single endothermic peak, attributed to a thermal transition (T_peak_ = 102.21 °C, ΔH_fusion_ = 99.606 J·g^−1^). The differences in onset temperatures between the IND-AcBz cocrystal and the individual components suggest the formation of a new phase: the IND-AcBz cocrystal it is observed in Table 4.

#### 2.3.2. Fourier Transformation Infrared Absorption Spectroscopy (FT-IR)

**Figure 8:**
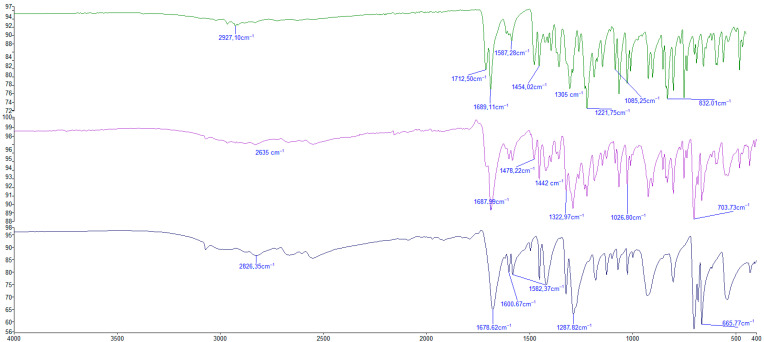
The FT-IR spectra of IND, cocrystal, and AcBz, in the wavenumber range (400–4000 cm^−1^).

The chemical structure of IND and the coformer (Figure 8) are characterized by similar chemical structures. In both cases, they have a carboxyl group through which hydrogen bonds are formed, generating a new molecular structure, the cocrystal [43].

The three spectra show overlapping absorptions due to the similarity of the structures. In spectrum 2, bands at 1687 cm^−1^ and 1290.20 cm^−1^ are observed, corresponding to the formation of a dimer of two carboxyl groups. It also corresponds to the region that is corresponding to the carboxylate (COO-). This overlap in the graphs in different solvents describes the binding of the co-former with the active molecule being present in the cocrystal graph, thus confirming the formation of the IND-AcBz cocrystal. IND is a polymorphic substance, and the gamma isoform was used in the preparation of these cocrystals [41]. It is characterized by differential absorption bands, compared to other forms in the 1600 cm^−1^ energy bands, indicating the carbon–carbon bond of the aromatic ring, in addition to the bands present at 1410 cm^−1^ and 1189 cm^−1^, which represent the hydroxyl group from the deformation of COOH and CH_2_ out of the plane, respectively [4]. On the other hand, other bands like the rest of the polymorphs are shown. Among the main bands is the CH_3_ group, with an absorption energy of 2927 cm^−1^, and C=O of the ketone, around 1690–1710 cm^−1^. This band is strong and intense, and clearly distinguishable from other bands such as the carboxylic group COOH, which is observed in two different bands at 1712.50 cm^−1^ (C=O) and 1221.75 cm^−1^ (OH). Along with the bands of the aromatic group 1600–1441 cm^−1^, another characteristic band of IND is observed, which corresponds to a chlorine bonded to an aromatic group in the energy band of 1085 cm^−1^, as shown in Table 5 [44].

Similarly, the other molecule used in the formation of the cocrystal, benzoic acid, has characteristic energy bands. Like IND, it has an aromatic group and a carboxylic acid. A strong band is observed at 1676.12 cm^−1^, indicating the presence of a carbonyl group. This, together with a broad band in the 3000–2200 cm^−1^ range and signals in the 1400–1100 cm^−1^ range—in this case, a strong band at 1287.82 cm^−1^—confirms the presence of a COOH group. Likewise, the range 3000–2200 cm^−1^ indicates the OH stretching bond of the acid. All this, combined with the bands at 704.21 cm^−1^ that belong to the aromatic group, defines the overall structure of benzoic acid [45,46,47]. Despite the similarities, differences between the three structures can be observed in the infrared spectrum.

In this study, 1:1 cocrystals are formed. This type of bond between benzoic acid and IND to form the cocrystal is characteristic and unique, creating hydrogen bonds between the carboxylic groups of both molecules. Figure 8 shows the bands at 1687 cm^−1^ and 1290.20 cm^−1^, which correspond to the H bonds.

Cocrystals are crystalline and are composed of two or more components in a stoichiometric ratio through non-covalent bonds. Cocrystals improve the physicochemical or mechanical properties of drugs (solubility, stability, flowability, and compressibility) without altering the chemical structure. The results obtained in this study confirm the successful formation of a cocrystal between IND and benzoic acid, as reflected in the unique DSC and IR patterns, distinct from the individual profiles of the pure components. Compared to previously reported cocrystals with other coformers, such as nicotinamide or fumaric acid, benzoic acid has proven to be an effective coformer, possibly due to its 1:1 interaction between the carboxylic groups of IND.

## 3. Materials and Methods

In this study, one different kind of cocrystals, formed by indomethacin (IND), was purchased from (Sigma-Aldrich, Steinheim, Germany) with benzoic acid (BzAc) (Roig Farma, S.A, Barcelona, Spain). All solvents (mass fraction purity > 0.995), chemicals (mass fraction purity > 0.990), and ethanol (Et) were from (Panreac AppliChem commercial, Barcelona, Spain), and ethyl acetate (EtAc) and methanol (Met) were from (Scharlkau, Barcelona, Spain). A thermoregulatory bath was used to determine the solubilities. After that, these cocrystals, as well as physical mixtures (PMs) and individual raw materials, were identified using a Differential Scanning Calorimeter (DSC) from (TA DSC 25, New Castle, DE, USA) Fourier Transform Infrared Absorption Spectroscopy (FT-IR) (Perkin-Elmer spectrum two, Shelton, CT, USA), Ultraviolet Diffraction (UV) from (PerkinElmer Lambda 365, Shelton, CT, USA), and solubility tests.

### 3.1. Indomethacin/Benzoic Acid Cocrystal Preparation

The cocrystals were prepared by a static solvent evaporation method. IND and the coformer, benzoic acid (AcBz), were added to flasks in specific concentration ratios, with a saturated solution of benzoic acid and varying amounts of IND in a 0 to 1 ratio. The solution was mixed under the selected solvent conditions—ethanol, methanol, or ethyl acetate—and stirred until the powder was completely dissolved. The flask was then shaken for 24 h at a constant temperature of 25 ± 0.04 °C, which was precisely maintained using thermoregulated baths and a thermostat. Equilibrium was considered to have been reached after 24 h, when the concentration variation between two consecutive samples taken at a two-hour interval was less than 2%. After reaching equilibrium, the samples were filtered with a 0.22 µm nylon filter. The filtered solution was then left to evaporate slowly in a fume hood at 25 °C for 24–72 h, to allow the cocrystals to form. Once the moisture was completely removed and the cocrystal powder was dry, it was collected. To quantify the IND and AcBz concentrations, UV spectrophotometry (PerkinElmer, Shelton, CT, USA) was used, and the final cocrystals were characterized by IR and DSC.

### 3.2. Solubility

The solubilities of IND, AcBz, and IND-AcBz in the different pure solvents, ethanol, methanol, and ethyl acetate, were all measured under the same conditions at a temperature of 25 °C (298.15 K), show in Table 6. The method involved adding successive and known amounts of the solid to a 100 mL flask, with constant stirring in a thermostatic bath. The flask was closed to prevent any solvent evaporation during the process.

#### Solubility Determination of Cocrystal of Indomethacin in Different Solvents

An excess amount of the sample is added to 10 mL of the solvent under study—ethanol, methanol, or ethyl acetate—and stirred for 4 h. The mixture is then placed in a thermostatic bath with agitation at 150 rpm and 25 °C for 24 h. Subsequently, it is filtered to obtain a clear saturated solution. The solution is diluted 20–50 times to allow for measurement of absorbance using a UV spectrophotometer (Peakin Lambda 365, Shelton, CT, USA). A calibration curve is prepared in the different solvents to enable the interpolation of concentration from the measured absorbance. Finally, the concentration of the original solution is calculated by multiplying it by the dilution factor.

The low solubility of IND is compensated for by the high solubility of benzoic acid in the solvent—100 times higher—allowing cocrystal formation under supersaturation conditions.

### 3.3. Indomethacin/Benzoic Acid Cocrystal Characterization

Compressive characterization of cocrystals requires elucidation of both intermolecular interactions and associated polymorphic behaviour.

#### 3.3.1. Differential Scanning Calorimeter (DSC)

A TA Instruments DSC calorimeter (TA DSC 25, New Castle, DE, USA) was used. The DSC analysis consisted of heating the samples of IND, ranging from 5 to 10 mg, where the temperature range was from 30 °C to 180 °C, at different heating rates of 5 and 10 °C/min.

#### 3.3.2. Fourier Transform Infrared Absorption Spectroscopy (FT-IR)

FT-IR spectra were obtained by using a spectrophotometer (Perkin-Elmer spectrum two, Shelton, CT, USA). An amount of 1–2 mg of samples was introduced directly into the detector. The spectrum was constructed from an average of 18 scans over a frequency range of 4000 to 400 cm^−1^, and the resolution was set to 1 cm^−1^.

## 4. Conclusions

In this study, we have successfully performed the crystallization of a cocrystal of IND with benzoic acid (IND–AcBz), a significant innovation, as this is the first time that this supramolecular association has been reported. The characterization by FT-IR and DSC unequivocally confirmed the formation of a new solid phase with 1:1 stoichiometry, in which benzoic acid interacts with the IND homodimer (carboxyl–carboxyl dimer) through hydrogen bonding.

The solvent evaporation method proved effective in producing stable cocrystals, as confirmed by spectroscopic and thermal analyses. Experimental data revealed a clear dependence of cocrystal solubility on the concentration of coformers, with ethanol showing the most favourable thermodynamic profile for cocrystallization formation. Mathematical models that explain the solubility behaviour of component concentrations in the solution considered the solubility product and the solution complexation. Based on these findings, IND-AcBz exhibits a significantly higher intrinsic solubility in ethanol, compared to pure IND. This finding demonstrates that co crystallization improves the biopharmaceutical properties of poor soluble APIs, but also emphasizes the effectiveness of benzoic acid as a cocrystallizer. Additionally, the application of PSDs proved to be a robust predictive tool for optimizing crystallization conditions. Our model, based on the total component concentrations and the complex formation constant, predicts solubility behaviour, proving a solid basis for the rational design of cocrystallization processes.

Findings offer valuable insights for the rational design of cocrystals, acutely for BCS Class II drugs such as IND, but also establishes a valuable predictive methodology. It is important to consider solvent selection, conformer ratio, and phase behaviour in optimization processes for enhanced solubility and bioavailability.

These advances represent a crucial step toward the design and development of new pharmaceutical formulations that optimize the efficacy and bioavailability of APIs. Future work should explore the in vivo performance of these systems and extend the modelling to multicomponent ternary systems.

## Figures and Tables

**Table 1:** Line regression analysis.

Solvent	Equation of Line	R^2^ Value	K_sp_ (M^2^)	K_11_ (M^−1^)
Ethanol	y=0.0052x+0.4794	0.96	5.2 × 10^−3^	92.19
Methanol	y=0.0038x−0.0168	0.99	3.8 × 10^−3^	0
Ethyl acetate	y=0.015x+0.0636	0.99	1.5 × 10^−2^	4.24

**Table 2:** IND/AcBz cocrystal solubility product.

Solvent	K_sp_ (M^2^)	K_11_ (M^−1^)
Ethanol	5.2 × 10^−3^	92.19
Methanol	3.8 × 10^−3^	0
Ethyl acetate	1.5 × 10^−2^	4.24

**Table 3:** Summary of cocrystal solubility and equilibrium at 25 °C: individual concentrations, total solubility, molar ratio, and Keu.

Solvent	[IND] (M)	[AcBz] (M)	[IND-AcBZ] (M)	[AcBz]/[IND]	[IND]_eu_ (M)	[AcBz] _eu_ (M)	K_eu_	S_cc_
Ethanol	0.01	1.02	0.47	101.50	0.05	0.16	3.30	0.09
Methanol	0.067	2.80	0	41.79	0.09	0.04	0.38	0.06
Etyl Acetate	0.23	1.77	0.0636	7.69	0.28	0.10	0.35	0.16

**Table 4:** Temperature and enthalpy of the solid phases and type of the transition derived from DSC.

Samples	DSC			Reported Melting Point	Ref
	T_onset (fusion)_/°C	T_peak(fusion)_/°C	ΔH_fusion_/Jg^−1^		
IND (γ form)	160.67	162.69	91.051	162	[41]
IND (α form)	-	-	-	154.5–155.5	[41]
Cocrystals EtOH	102.21	105.25	99.606	-	
Cocrystal MeOH	99.87	105.18	100.13	-	
Cocrystal EtAc	109.85	116.65	82.60	-	
Benzoic Acid	122.75	125.53	124.20	122	[42]

**Table 5:** FTIR signals associated with IND, cocrystal, and AcBz.

IND	Cocrystal	AcBz	Assigned Functional Group
2927.10		2826.37	W (OH)
	2635		W (C=O modified)
1713	1687	1687.12	S (COOH)
1689			S (C=O amide)
		1600	W (C=C)
1587		1582	W (c=c) aromatic
1454	1442	1452	M (C=C) aromatic
1305	1323		M (C-H in plane)
1221		1287	S (C-O)
	1028		m (C-C in plane)
1085			m(C-Cl)
832			C-H out of plane
	703		Ring deformation
		665	C-H out of plane

m, S, W stand for medium, strong, and weak, for stretching, deformation, and torsional, respectively.

**Table 6:** The solubilities of IND, AcBz, and IND-AcBz in the different pure solvents, ethanol, methanol, and ethyl acetate.

Sample	Ethanol (M)	Methanol (M)	Etyl Acetate (M)
AcBz	1.015	2.800	1.77
IND	0.010	0.067	0.23
IND/AcBz (1:1)	0.089	0.056	0.16

## Data Availability

The raw data supporting the conclusions of this article will be made available by the authors on request.
